# Correction to “Evaluating the Efficacy of Hyperbaric Oxygen Therapy for Acute Carbon Monoxide Poisoning: A Systematic Review and Meta‐Analysis”

**DOI:** 10.1002/ams2.70162

**Published:** 2026-09-14

M. Fujita , M. Todani , Y. Ajimi , et al., “Evaluating the Efficacy of Hyperbaric Oxygen Therapy for Acute Carbon Monoxide Poisoning: A Systematic Review and Meta‐Analysis,” Acute Medicine & Surgery 13, no. 1 (2026): e70114, 10.1002/ams2.70114.41624627 PMC12853008

M. Fujita , M. Todani , Y. Ajimi , et al., “Response to Comments on “Evaluating the Efficacy of Hyperbaric Oxygen Therapy for Acute Carbon Monoxide Poisoning”,” Acute Medicine & Surgery 13, no. 1 (2026): e70147, 10.1002/ams2.70147.42436671 PMC13354944

The name of the ninth author was misspelled and has been updated from Tetsuya Samkamoto to Tetsuya Sakamoto.

The online versions of these articles have been corrected accordingly.

We apologize for this error.

